# Correction: p16 deficiency attenuates intervertebral disc degeneration by adjusting oxidative stress and nucleus pulposus cell cycle

**DOI:** 10.7554/eLife.112978

**Published:** 2026-08-21

**Authors:** Hui Che, Jie Li, You Li, Cheng Ma, Huan Liu, Jingyi Qin, Jianghui Dong, Zhen Zhang, Cory J Xian, Dengshun Miao, Liping Wang, Yongxin Ren

**Keywords:** Human, Mouse

Che H, Li J, Li Y, Ma C, Liu H, Qin J, Dong J, Zhang Z, Xian CJ, Miao D, Wang L, Ren Y. 2020. p16 deficiency attenuates intervertebral disc degeneration by adjusting oxidative stress and nucleus pulposus cell cycle. *eLife* **9**:e52570. doi: 10.7554/eLife.52570.

Published 3 March 2020

We are publishing this correction to address a transcription error in the reported catalog number for the p16 antibody used in our study.

During manuscript preparation, the catalog number for the anti-p16 antibody was incorrectly listed as ab51243 (RRID:AB_2059963), which is specific for p16-ARC. The actual antibody used in all our experiments was the anti-CDKN2A/p16INK4a antibody [EPR20418] (Abcam, Cat# ab211542, RRID:AB_2891084).

In support of the correction, the original procurement records (sales contract, invoice, and receipt) demonstrating the purchase of the correct antibody (ab211542) were shared and reviewed by the editors.

This correction affects two locations of the Materials and methods section of the published article:

1. In the "Key resources table”:

Corrected text:

Antibody | Anti-CDKN2A/p16INK4a antibody (rabbit monoclonal) [EPR20418] | Abcam | Cat# ab211542, RRID:AB_2891084 | IF (1:100); WB (1:1000)

Original text:

Antibody | Anti-p16 ARC antibody (rabbit monoclonal) | Abcam | Cat# ab51243, RRID:AB_2059963 | IF (1:100); WB (1:1000)

2. In the "Immunofluorescence" section:

Corrected text:

Then, the cells were treated with primary antibody against p16 (ab211542, Abcam, Cambridge, UK) for one night at 4 °C...

Original text:

Then, the cells were treated with primary antibody against p16 (ab51243, Abcam, Cambridge, UK) for one night at 4 °C...

We apologize for this oversight. This correction does not change the experimental data, results, or conclusions of the original article.

