# Supplementary material for: p16 deficiency attenuates intervertebral disc degeneration by adjusting oxidative stress and nucleus pulposus cell cycle
Source: eLife. 2020 Mar 3;9:e52570. doi: 10.7554/eLife.52570 (PMC7065909; doi:10.7554/eLife.52570)
Supplement: Supplementary file 1. [file elife-52570-supp1.docx]

**Supplementary File 1** Primer sequences for CHIP

| **Binding site** | **Score** | **Primer Sequence** | **Length (bp)** |
| --- | --- | --- | --- |
| GGGGAATGCT | 4.03 | 5`CCTAATGCCCCCCTACAGAG | 221 |
|  |  | 3`TTTCAGGCACCCTCATACCA |  |
| GGTAAATTCT | 4.46 | 5`GGGAGAAAGAGCAGCCATAC | 201 |
|  |  | 3`ACAAACCCAAGACAAAACGG |  |
| CTCACTTTCC | 5.27 | 5`GGAAAGAGAAGTCTGCCGCT | 91 |
|  |  | 3`TACAGGGGAGGAAAGTGAGG |  |
| GGGGCTTGAC | 5.07 | 5`TCCTCCCCTGTAAGGTCTGG | 131 |
|  |  | 3`TCTCAGTGGCTTCCTGTTCA |  |
| TCGACTTCCC | 5.68 | 5`GAACAGGAAGCCACTGAGA | 331 |
|  |  | 3`GCAGAAAGGAGGGGTGAGTC |  |
