# Supplementary material for: p16 deficiency attenuates intervertebral disc degeneration by adjusting oxidative stress and nucleus pulposus cell cycle
Source: eLife. 2020 Mar 3;9:e52570. doi: 10.7554/eLife.52570 (PMC7065909; doi:10.7554/eLife.52570)
Supplement: Supplementary file 2. [file elife-52570-supp2.docx]

**Supplementary File 2** Patients information

| NO. | Gender | Age | Pfirrmann score | NO. | Gender | Age | Pfirrmann score |
| --- | --- | --- | --- | --- | --- | --- | --- |
| 1 | F | 54 | 2 | 9 | F | 62 | 3 |
| 2 | F | 43 | 2 | 10 | M | 59 | 3 |
| 3 | M | 36 | 2 | 11 | M | 47 | 3 |
| 4 | M | 53 | 2 | 12 | F | 37 | 3 |
| 5 | M | 42 | 2 | 13 | F | 63 | 3 |
| 6 | F | 53 | 2 | 14 | M | 55 | 3 |
| 7 | M | 57 | 3 | 15 | M | 63 | 3 |
| 8 | F | 65 | 3 | 16 | M | 43 | 3 |
| NO. | Gender | Age | Pfirrmann score | NO. | Gender | Age | Pfirrmann score |
| 17 | M | 61 | 3 | 25 | M | 63 | 4 |
| 18 | F | 54 | 4 | 26 | M | 73 | 5 |
| 19 | F | 59 | 4 | 27 | F | 48 | 5 |
| 20 | M | 72 | 4 | 28 | F | 59 | 5 |
| 21 | M | 78 | 4 | 29 | F | 66 | 5 |
| 22 | M | 55 | 4 | 30 | M | 71 | 5 |
| 23 | F | 64 | 4 | 31 | M | 76 | 5 |
| 24 | M | 52 | 4 | 32 | F | 73 | 5 |
| NO. (Number); M (male); F (femal) | | | | | | | |
