# Supplementary material for: p16 deficiency attenuates intervertebral disc degeneration by adjusting oxidative stress and nucleus pulposus cell cycle
Source: eLife. 2020 Mar 3;9:e52570. doi: 10.7554/eLife.52570 (PMC7065909; doi:10.7554/eLife.52570)
Supplement: Supplementary file 3. [file elife-52570-supp3.docx]

**Supplementary File 3** Primer sequences for RT-PCR

| **Name** | **Sequence** | **Length (bp)** |
| --- | --- | --- |
| TNF-α | 5`CCTGTAGCCCACGTCGTAG | 148 |
|  | 3`GGGAGTAGACAAGGTACAACCC |  |
| IL-1β | 5`GCAACTGTTCCTGAACTCAACT | 89 |
|  | 3`ATCTTTTGGGGTCCGTCAACT |  |
| IL-6 | 5`TAGTCCTTCCTACCCCAATTTCC | 76 |
|  | 3`TTGGTCCTTAGCCACTCCTTC |  |
| MMp3 | 5`ACATGGAGACTTTGTCCCTTTTG | 192 |
|  | 3`TTGGCTGAGTGGTAGAGTCCC |  |
| MMp9 | 5`CTGGACAGCCAGACACTAAAG | 144 |
|  | 3`CTCGCGGCAAGTCTTCAGAG |  |
| MMp10 | 5`GAGCCACTAGCCATCCTGG | 101 |
|  | 3`CTGAGCAAGATCCATGCTTGG |  |
| MMp13 | 5`CTTCTTCTTGTTGAGCTGGACTC | 173 |
|  | 3`CTGTGGAGGTCACTGTAGACT |  |
| Gpx1 | 5`CAATCAGTTCGGACACCAGGAG | 128 |
|  | 3`TCTCACCATTCACTTCGCACTTC |  |
| Gpx3 | 5`CTTCTTCTTGTTGAGCTGGACTC | 173 |
|  | 3`CTGTGGAGGTCACTGTAGACT |  |
| CAT | 5`AGCGACCAGATGAAGCAGTG | 180 |
|  | 3`TCCGCTCTCTGTCAAAGTGTG |  |
| SOD1 | 5`GGTGAACCAGTTGTGTTGTC | 203 |
|  | 3`CCGTCCTTTCCAGCAGTC |  |
| SOD2 | 5`CAGACCTGCCTTACGACTATGG | 112 |
|  | 3`CTCGGTGGCGTTGAGATTGTT |  |
| Aggrecan | 5`GGTGAACCAGTTGTGTTGTC | 203 |
|  | 3`CCGTCCTTTCCAGCAGTC |  |
| Collagen Ⅱ | 5`GGGAATGTCCTCTGCGATGAC | 65 |
|  | 3`GAAGGGGATCTCGGGGTTG |  |
| GAPDH | 5`GGTCGGTGTGAACGGATTTG | 508 |
|  | 3`ATGAGCCCTTCCACAATG |  |
